# Human T-lymphotropic viruses (HTLV) in England and Wales, 2004 to 2013: testing and diagnoses

**DOI:** 10.2807/1560-7917.ES.2017.22.21.30539

**Published:** 2017-05-25

**Authors:** Georgina Ireland, Sara Croxford, Jennifer Tosswill, Rajani Raghu, Katy Davison, Patricia Hewitt, Ruth Simmons, Graham Taylor

**Affiliations:** 1 National Infection Service, Public Health England, London, United Kingdom; 2 Microbiology Services, NHS Blood and Transplant, London, United Kingdom; 3 Section of Virology, Imperial College London, London, United Kingdom

**Keywords:** HTLV-1, HTLV-2, Testing, Diagnosis, Surveillance, Epidemiology

## Abstract

Human T-lymphotropic virus (HTLV) infection has been under enhanced surveillance in England and Wales since 2002, however, little is known about testing patterns. Using data from two surveillance systems held at Public Health England, we described HTLV antibody testing patterns between 2008 and 2013 and the demographic and clinical characteristics of persons diagnosed with HTLV in England and Wales between 2004 and 2013. An increase in HTLV testing was observed in England between 2008 and 2013 (3,581 to 7,130). Most tests (82%; 7,597/9,302) occurred within secondary care, 0.5% (48/9,302) of persons were reactive for HTLV antibodies and 0.3% (27/9,302) were confirmed positive. Increasing age and female sex were predictors of a reactive HTLV screen and confirmed diagnosis. Testing in primary care including sexual health and antenatal services was infrequent. Between 2004 and 2013, 858 people were diagnosed with HTLV, most of whom were female (65%; 549/851), of black Caribbean ethnicity (60%), not born in the United Kingdom (72%; 369/514) and asymptomatic at diagnosis (45%; 267/595). Despite increased testing, the epidemiology and clinical features of those diagnosed with HTLV have remained consistent. Apart from donor screening, testing for HTLV infection remains uncommon, except to diagnose associated disease.

## Introduction

The human T-lymphotropic viruses (HTLV), discovered in the early 1980s [1,2], have now infected an estimated 10 million people worldwide [3]. HTLV-1 is endemic in many tropical and subtropical regions, particularly the Caribbean, Iran, Melanesia, South America, southern India, southern Japan and West Africa [3]. In endemic areas, the distribution of infection varies, with seroprevalence among adults ranging between 0.1% and 30% [4]. In Europe and North America, HTLV-1 is predominately found among persons migrating from endemic areas, while HTLV-2 has been associated with injecting drug use [4].

Although the majority (ca 90%) of HTLV-1-infected individuals remain asymptomatic carriers, the other 10% will develop one or more of several diseases [5]: 2–6% will develop adult T-cell leukaemia/lymphoma (ATLL), a highly aggressive T-cell malignancy, while 2–3% develop a variety of chronic inflammatory syndromes, most notably HTLV-1-associated myelopathy (HAM)/tropical spastic paraparesis (TSP) [6]. Other symptoms associated with HTLV infection include uveitis, thyroiditis, alveolitis, polymyositis and an impairment of immunity most strikingly associated with risk of strongyloidiasis observed in those HTLV-1 carriers exposed to *Strongyloides stercoralis*. There is no vaccine or effective treatment to reduce or eliminate HTLV viral load and treatments available for those who have malignant or inflammatory manifestations of HTLV infection are limited.

In endemic areas, HTLV is predominantly acquired through mother-to-child transmission (MTCT), primarily through breastfeeding [7], and through sexual contact, with male-to-female transmission approximately four times more likely to occur than female-to-male transmission [8]. However, there are no routine antenatal screening programmes in Europe. Blood transfusion has also been an important source of HTLV infection in some endemic areas, especially in Japan [4]. Blood, tissue and stem cell donations are currently routinely screened in a number of countries, including the United Kingdom (UK), while some blood services perform selective screening of first-time donors or rely on leucodepletion as an HTLV risk-reduction measure [9]. Other blood services, including many in Europe, have no screening programmes.

HTLV-1 testing was introduced in the UK in 1986 and routine testing of all blood donations was introduced in 2002 [10]. Surveillance by Public Health England (PHE; previously Health Protection Agency) was introduced in 1987 and enhanced surveillance in 2002 [11]. It is estimated that more than 22,000 persons are infected with HTLV in England and Wales [12] and the seroprevalence in pregnant women in the UK is 0.3 per 1,000 [13]. However, there is little information on patterns of HTLV testing [14], which will influence the rate of undiagnosed HTLV. Here we examine the patterns of HTLV antibody testing in England from 2008 to 2013, with data from the Sentinel Surveillance of Blood Borne Virus Testing (SSBBV), along with HTLV diagnoses in England and Wales over the 10-year period between 2004 and 2013 collected through the enhanced surveillance of new diagnoses of HTLV.

## Methods

### Data sources

**Figure 1:**
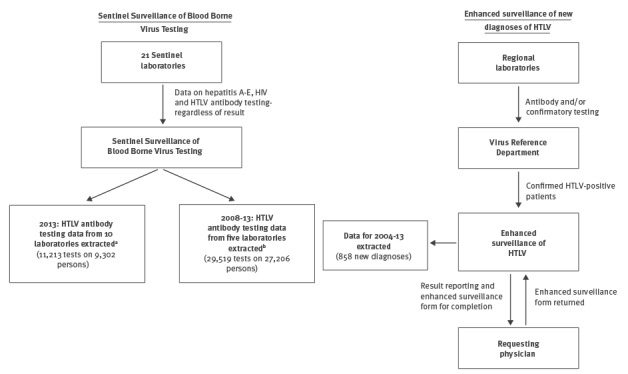
Flow of data within the Sentinel Surveillance of Blood Borne Virus Testing, England, 2008–2013, and the enhanced surveillance of HTLV, England and Wales, 2004–2013

HIV: human immunodeficiency virus; HTLV: human T-lymphotropic virus.

^a^ Laboratories offering testing in 2013.

^b^ Laboratories testing for HTLV throughout the period 2008 to 2013.

Two data sources were used for our analysis: (i) SSBBV, to look at HTLV antibody testing in 2013 and changes in testing between 2008 and 2013, and (ii) the enhanced surveillance of new diagnoses of HTLV to describe the epidemiological and clinical characteristics of persons diagnosed with HTLV between 2004 and 2013 (Figure 1).

SSBBV collects information on tests for hepatitis A–E, HIV and HTLV, regardless of result, from 21 participating sentinel laboratories in England. Individuals are de-duplicated and linked to all their test results, using a combination of coded surname (soundex), first initial, date of birth, National Health Service (NHS) number and clinic number [15]. Alongside the test result, SSBBV records demographic information and the service requesting the test. Information on HTLV antibody testing has been collected by SSBBV since 2005, with 10 laboratories offering HTLV antibody testing in 2013, five of whom were testing for HTLV throughout the period 2008 to 2013. Coverage varies annually, but in 2013, SSBBV captured front line testing for these viruses by laboratories covering ca 40% of the general population of England and is broadly representative of laboratories providing routine and reference testing.

Enhanced surveillance of new diagnoses of HTLV in England and Wales has been conducted since 2002 by PHE [12,16]. In brief, blood samples are sent to the Virus Reference Department (VRD) in PHE Colindale for either antibody and/or confirmatory testing, by Western blot or PCR. Laboratory case reports of confirmed positive samples are provided to the HIV/sexually transmitted infection (STI) Surveillance Unit, who send an enhanced surveillance form to the referring clinician. The data collected include supplementary epidemiological and clinical information, including probable route of HTLV exposure, ethnicity, country of birth, probable country of infection and data on clinical symptoms at diagnosis. Enhanced forms are also received from NHS Blood and Transplant/PHE Epidemiology Unit for England and Wales when blood donors are found positive on routine donation screening. All data collected by PHE are pseudo-anonymised, with a soundex code and first initials collected instead of full names. Death reports on individuals who died from an HTLV-related illness are received from the Office of National Statistics and matched to the HTLV new diagnosis database using soundex code, date of birth and sex.

### HTLV testing in 2013

Demographic and testing data for all individuals tested for HTLV antibodies in 2013 from 10 participating centres in England were extracted from SSBBV to give a snapshot of HTLV testing, as 2013 had the highest number of laboratories providing HTLV antibody tests for a full year. Where individuals had been tested more than once for HTLV antibodies, a reactive test was recorded over any negative or equivocal tests. Where all tests within 2013 were negative or equivocal, information from the most recent test was used.

### Trends in HTLV testing: 2008 to 2013

To investigate trends, testing data for the period 2008–13 from the five sentinel laboratories with complete testing data for the 6-year period was used. Demographic and testing data for all individuals tested for HTLV antibodies between January 2008 and December 2013 were extracted from SSBBV. A cohort approach was used, with each individual recorded only once in each year tested. Where individuals had been tested more than once for HTLV antibodies in a year, a reactive test was recorded over any negative or equivocal tests. Where all tests in a year were negative or equivocal, information from the most recent test was used. Individuals who were reactive for HTLV antibodies were excluded from appearing in subsequent testing year cohorts.

### HTLV testing in 2013 and trends in HTLV testing: 2008 to 2013

In both datasets, tests were linked to information on the location of the service requesting the test. Age at test was calculated using date of birth. Individuals younger than 1 year were excluded from the analysis, as a reactive result at that age may reflect maternal HTLV antibody status. Individuals with a reactive HTLV antibody test were considered to have confirmed HTLV-1 or HTLV-2 infection if a Western blot or PCR performed at the VRD at PHE, which provides the confirmatory HTLV diagnostics, was positive. Requesting services were grouped into primary care (which included testing in general practice (GP), genitourinary medicine (GUM), accident and emergency (A and E), occupational health, prisons and drug dependency units) and secondary care services, along with an additional category called ‘other’, which included tests requested by blood and stem cell banks, research studies and where requester information was not available.

### Enhanced surveillance of new HTLV diagnoses: 2004 to 2013

Data for the years 2004 to 2013 were used for these analyses, based on reports received by end of October 2015. Numbers may rise as further reports are received, particularly for recent years.

### Statistical analysis

Unless otherwise stated, HTLV in this paper refers to both HTLV-1 and HTLV-2. Stata SE (Version 13.1) was used, with Wilcoxen rank-sum tests to compare continuous variables and chi-squared and Fisher’s exact tests for categorical variables. A multivariate logistic regression, adjusted for age, sex, service type and year of test, was used to look at predictors of a reactive HTLV antibody test and confirmed HTLV diagnoses in those tested between 2008 and 2013. This dataset was used as more people were tested between 2008 and 2013 compared with the 2013 only dataset.

Joinpoint regression analysis was used to analyse changes in testing and positivity rates between 2008 and 2013, using Joinpoint Software (Version 4.3.1.0). Joinpoint identifies significant increases and decreases in testing and positivity rates over time, and whether there was a point at which a statistically significant change in the slope occurred during the period. The annual average percent change (APC) in testing numbers, with 95% confident intervals (95% CI), are reported. All proportions reported are excluding unknowns, and tests were considered statistically significant if the p value was lower than 0.05.

## Results

### HTLV testing in 2013

During 2013, 10 laboratories participating in sentinel surveillance reported HTLV testing in England, with 11,213 HTLV antibody tests conducted on 9,302 persons. Median age at testing was 45 years (interquartile range (IQR): 31–58 years), and men were older than women (49 vs 40 years; p < 0.001) (Table 1).

**Table 1:** Characteristics of persons testing for HTLV in 10 sentinel laboratories, England, 2013 (n = 9,302)

Patient characteristics	n	Reactive		Confirmed positive	
		n	%	n	%
*Individuals*	9,302	48	0.5	27	0.3
*Sex*					
Male	4,562	22	0.5	10	0.2
Female	4,312	26	0.6	17	0.4
Unknown	428	0	0.0	0	0.0
*Age (years)*					
1–29	1,901	8	0.4	2	0.1
30–44	2,528	8	0.3	2	0.1
45–59	2,480	22	0.9	16	0.6
≥ 60	2,094	10	0.5	7	0.3
Unknown	299	0	0.0	0	0.0
*Service type*					
Primary	862	4	0.5	4	0.5
Secondary	7,597	39	0.5	22	0.3
Other	843	5	0.6	1	0.1

HTLV: human T-lymphotropic virus.

The majority (81.7%, 7,597/9,302) were tested within secondary care. Overall, the most common settings were haematology services (18.1%, 1,680/9,302), specialist renal services (13.4%, 1,250/9,302) and general medical or surgical departments (9.9%, 921/9,302). Among primary care settings, the most common for HTLV testing was GP with 265 tests (2.8% of all testing), followed by GUM services (2.7%, n = 247). In 2013, women’s services requested just 62 tests (0.7%), comprising 22 HTLV tests within antenatal care, 12 in gynaecological services, 18 in birthing and obstetric services and 10 in postnatal services. During 2013, there were 664,517 live births in England [17].

Overall, 0.5% (n = 48) of persons tested were reactive for HTLV antibodies in 2013. HTLV antibody-reactive women were older than men (44 vs 55 years; p < 0.001), and the proportion of reactive tests was highest in persons aged 45–59 years. More than half (n = 27) of those reactive for HTLV were confirmed HTLV-positive by the VRD at PHE. The age and sex distribution of those with a confirmed HTLV-positive result was similar to those reactive to HTLV antibodies.

### Trends in HTLV testing: 2008 to 2013

**Figure 2:**
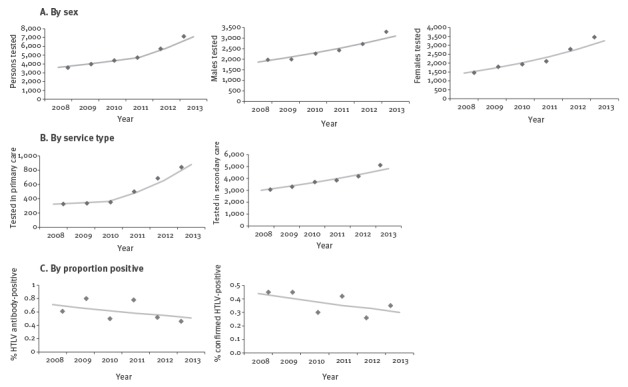
Trends in HTLV antibody testing in five sentinel laboratories, England, 2008–2013 (n = 29,519)

Five sentinel laboratories consistently reported HTLV testing each year between 2008 and 2013, with 29,519 HTLV antibody tests performed on 27,206 persons (13,316 in men and 12,558 in women, of those for whom sex was known). Overall, the number of persons tested increased by an average of 9.5% (95% CI: 7.4–11.7) annually between 2008 and 2011, and by 22.2% (95% CI: 17.5–27.2) annually between 2011 and 2013 (Figure 2 and Table 2).

HTLV: human T-lymphotropic virus.

**Table 2:** Demographic characteristics and adjusted odds ratio of being reactive to HTLV antibodies and having a confirmed HTLV diagnosis, five participating sentinel centres, England, 2008–2013 (n = 29,519)

Patient characteristics	All tested **(n = 29,519)**	Reactive HTLV screen				Confirmed HTLV diagnosis			
		Number (n = 176)	aOR	95% CI	p value	Number (n = 89)	aOR	95% CI	p value
*Age (aOR/10 year increase)*	*45 (33–59)* ^a^	*52 (40–63)* ^a^	*1.2*	*1.1–1.3*	*0.001*	*53 (42–64)* ^a^	*1.4*	*1.2–1.5*	*< 0.001*
*Sex*									
Male	14,653	73	1	Ref		30	1	Ref	
Female	13,524	102	1.7	1.2–2.3	0.001	59	2.4	1.6–3.8	< 0.001
Not reported^b^	1,342	1	NA			0	NA		
*Service type*									
Primary	3,043	15	1	Ref		12	1	Ref	
Secondary	23,142	152	1.2	0.7–2.0	0.6	75	0.6	0.3–1.2	0.1
Other	3,334	9	0.5	0.2–1.1	0.1	2	0.1	0.03–0.5	0.006
*Year tested*									
2008	3,581	22	1	Ref		15	1	Ref	
2009	3,977	32	1.3	0.8–2.3	0.3	14	0.9	0.4–1.9	0.8
2010	4,393	22	0.8	0.4–1.5	0.5	9	0.5	0.2–1.1	0.1
2011	4,719	37	1.3	0.8–2.2	0.3	16	0.9	0.4–1.8	0.7
2012	5,719	30	0.9	0.5–1.6	0.7	14	0.6	0.3–1.3	0.2
2013	7,130	33	0.8	0.5–1.4	0.5	21	0.8	0.4–1.6	0.5

aOR: adjusted odds ratio; CI: confidence interval; HTLV: human T-lymphotropic virus; NA: not applicable; Ref: reference value.

^a^ Median and interquartile ranges.

^b^ Persons of unknown sex were not included in the logistic regression.

The number tested each year between 2008 and 2013 increased by 17.8% (95% CI: 12.3–23.5) in women and by 10.7% (95% CI: 6.8–14.7) in men (Figure 2). Overall, the median age at test decreased from 47 years in 2008 to 43 years in 2013 (p < 0.001) and from 44 years to 38 years among women (p < 0.001); there was no significant change for men. While the number of persons screened for HTLV infection in primary care settings increased 157% overall, a result of more testing requested within GP and occupational health services, the APC was not significant (2008–10: 5.6%, 95% CI: −53.8 to 141 and 2010–13: 34.4%, 95% CI: −11.1 to 103). The number of people tested within secondary care increased 9.9% (95% CI: 6.7 to 13.2) annually between 2008 and 2013.

In the five sentinel laboratories, 0.6% of persons tested (n = 176) had a reactive HTLV antibody result between 2008 and 2013, with no significant difference in the proportion positive by year (p = 0.2). All other variables remained stable. The specialities that found the most number of persons reactive for HTLV were haematology (n = 53) and general medical and surgical wards (n = 20). Among those with an initially reactive HTLV antibody result, 50.6% (n = 89) were subsequently confirmed to have HTLV, a prevalence of 0.3%.

### Predictors of a reactive HTLV screen and confirmed HTLV diagnosis

Using a multiple logistic regression (Table 2), the odds of testing reactive for HTLV antibodies did not change by year or between primary and secondary care, but increased for each 10-year increase in age (odds ratio (OR) = 1.2; 95% CI: 1.1–1.3) and for women compared with men (OR = 1.7; 95% CI: 1.2–2.3). These findings were similar for a confirmed HTLV diagnosis (Table 2), although the odds of being confirmed following a test in ‘other services’ (i.e. not specified) were lower than in primary care (OR = 0.1; 95% CI: 0.03–0.5).

### HTLV diagnoses in England from enhanced surveillance 2004 to 2013

Between 2004 and 2013, 858 new HTLV diagnoses were reported to PHE in England and Wales, averaging 86 cases per year (range: 76–98) (Table 3).

**Table 3:** Demographic and clinical characteristics of persons reported to the enhanced surveillance of HTLV programme, England and Wales, 2004–2013 (n = 858)

Patient characteristics	n	%
*Individuals*	858	100.0
*Sex*		
Male	302	35.2
Female	549	64.0
Unknown	7	0.8
*Age (years)*		
1–29	81	9.4
30–44	183	21.3
45–59	308	35.9
≥ 60	284	33.1
Unknown	2	0.2
*Ethnicity*		
White	102	11.9
Black Caribbean	328	38.2
Black African	66	7.7
Black other	7	0.8
Indian/Pakistani/Bangladeshi	19	2.2
Other/mixed	27	3.1
Unknown	309	36.0
*HTLV type*		
HTLV-1	773	90.1
HTLV-2	40	4.7
Unknown	45	5.2
*Exposure*		
Heterosexual sex and/or mother-to-child transmission	365	42.5
Other ^a^	36	4.2
Unknown	457	53.3
*HTLV-associated symptoms*		
Asymptomatic	267	31.1
HTLV symptoms	193	22.5
Non-HTLV symptoms	135	15.7
Unknown	263	30.7
*Year of diagnosis*		
2004–08	406	47.3
2009–13	452	52.7
*HIV co-infection*	43	5.0
*Deaths*	96	11.2

HIV: human immunodeficiency virus; HTLV: human T-lymphotropic virus.

^a^ Includes transfused blood, sex between men and people who inject drugs.

Where reported, more women were diagnosed with HTLV than men (64.5%; 549/851). Median age at diagnosis was 52 years (IQR: 42–66), with no difference between men and women (50 vs 53 years; p = 0.2). The majority of diagnoses were among persons of black Caribbean ethnicity (59.7%, 328/549), followed by white (18.6%, n = 102), black African (12.0%, n = 66), other/mixed (4.9%, n = 27), south Asian (3.5%, n = 19) and black other ethnicities (1.3%, n = 7). When compared with white individuals, persons of other/mixed and South Asian ethnicity were younger (49 vs 36 and 29 years, respectively; both p < 0.05), whereas persons of black Caribbean ethnicity were older (49 vs 53 years; p < 0.002). Where country of birth was reported (59.9%, n = 514), the majority of HTLV-positive persons of black African (51/56), black Caribbean (231/284), South Asian (14/17) and other/mixed ethnicity (17/25) were born abroad. Overall, 5.0% of persons diagnosed with HTLV (n = 43) were co-infected with HIV.

The majority of HTLV infections diagnosed, where type was reported, were HTLV-1 (95.1%, 773/813) and only 4.9% (n = 40) of persons were infected with HTLV-2. Most persons diagnosed with HTLV-1 were of black Caribbean origin (62.8%, 319/508 with known ethnicity), whereas most persons with HTLV-2 were of white ethnicity (n = 18).

Where reported (62.5%, n = 536), the most common reason for testing for HTLV was clinical symptoms (43.7%, n = 234), followed by blood donation (27.1%, n = 145). Persons diagnosed following blood donation were younger than those tested because of symptoms (41 vs 56 years; p < 0.001) and a higher proportion of women tested positive in the context of blood donation than men (30.7%, 109/355 v 20.2%, 36/178; p = 0.01). There was no difference by sex in the proportion of persons presenting with symptoms (41.7%, 148/355 vs 47.8%, 85/178; p = 0.1). Persons of black ethnicity (54.9%, 200/364) and those diagnosed with HTLV-1 (45.6%, 226/496) were most commonly diagnosed following the presentation of symptoms, whereas persons of white (46/98), other/mixed (13/23) and South Asian ethnicity (14/19) and those with HTLV-2 (10/19) were most commonly diagnosed when tested in the context of blood donation.

Probable route of infection was reported for 46.7% (n = 401) of persons between 2004 and 2013. Among those, just over a quarter were probably infected through heterosexual sex (27.7%, n = 111), another quarter (25.2%, n = 101) via MTCT, and 38.2% (n = 153) reported both MTCT and heterosexual contact. MTCT and heterosexual contact were the most common routes of probable infection regardless of ethnicity or sex, but men were more likely to report other transmission routes than women (16.1% vs 5.3%; p < 0.001) and almost all (9/10) persons with a probable infection following injecting drug use were of white ethnicity. A smaller proportion of persons infected with HTLV-2 reported a probable infection route of heterosexual contact and/or MTCT than those with HTLV-1 (57.9% vs 92.6%; p < 0.001), with injecting drug use accounted for the remaining infection in HTLV-2 (n = 8).

The majority of persons with symptoms reported were asymptomatic (44.9%, 267/595), 20.5% (n = 122) had ATLL, 15.0% (n = 89) had other or non-HTLV related symptoms, 11.9% (n = 71) had TSP/HAM and 7.7 (n = 46) had other malignancies/neurology (Table 4).

**Table 4:** Demographic and clinical characteristics of persons reported to the enhanced surveillance of HTLV programme, by symptoms, England and Wales, 2004–2013

Patient characteristics	Asymptomatic		ATLL		TSP/HAM		Other HTLV symptoms		Non-HTLV symptoms		Not reported	
	n	%	n	%	n	%	n	%	n	%	n	%
*Individuals*	267		122		71		97		38		263	
*Median age (IQR)*	44 (33–54)		59 (50–69)		53 (46–68)		60 (46–73)		57 (47–72)		55 (43–68)	
*Sex*												
Male	85	31.8	42	34.4	19	26.8	40	41.2	17	44.7	99	37.6
Female	181	67.8	79	64.8	52	73.2	57	58.8	21	55.3	159	60.5
Not Reported	1	0.4	1	0.8	0	0.0	0	0.0	0	0.0	5	1.9
*Ethnicity*												
White	74	27.7	3	2.5	7	9.9	5	5.2	5	13.2	8	3.0
Black Caribbean	117	43.8	77	63.1	44	62.0	45	46.4	23	60.5	22	8.4
Black African	31	11.6	10	8.2	10	14.1	8	8.2	4	10.5	3	1.1
Black Other	3	1.1	3	2.5	0	0.0	1	1.0	0	0.0	0	0.0
Indian/Pakistani/Bangladeshi	16	6.0	0	0.0	1	1.4	1	1.0	0	0.0	1	0.4
Other/Mixed	18	6.7	1	0.8	0	0.0	2	2.1	2	5.3	4	1.5
Not Reported	8	3.0	28	23.0	9	12.7	35	36.1	4	10.5	225	85.6
*Exposure*												
Heterosexual sex and/or mother to child transmission	189	70.8	54	44.3	45	63.4	38	39.2	25	65.8	14	5.3
Other ^a^	26	9.7	1	0.8	1	1.4	4	4.1	2	5.3	2	0.8
Not Reported	52	19.5	67	54.9	25	35.2	55	56.7	11	28.9	247	93.9
*HTLV type*												
HTLV-I	241	90.3	120	98.4	68	95.8	87	89.7	32	84.2	225	85.6
HTLV-II	16	6.0	0	0.0	0	0.0	4	4.1	3	7.9	17	6.5
Unknown	10	3.7	2	1.6	3	4.2	6	6.2	3	7.9	21	8.0
*HIV Co-infection*	16	6.0	5	4.1	2	2.8	8	8.2	6	15.8	6	2.3
*Deaths*	4	1.5	53	43.4	3	4.2	17	17.5	4	10.5	15	6.7

ATLL: T-cell leukaemia/lymphoma; HAM/TSP: HTLV-1-associated myelopathy/tropical spastic paraparesis; HTLV: human T-lymphotropic virus.

^a^ Includes blood transfusion, sex between men and people who inject drugs.

Persons who were asymptomatic at diagnosis were younger than persons with ATLL and TSP/HAM (asymptomatic: 44 years vs ATLL: 55 years and TSP/HAM: 53 years, both p < 0.001). Regardless of ethnicity, most persons were asymptomatic at diagnosis, but almost all persons with ATLL were of black ethnicity (90/94). Where reported, a higher proportion of persons with HTLV-2 were asymptomatic than with HTLV-1 (69.6% vs 44.0%; p = 0.02).

Of all persons diagnosed between 2004 and 2013 in England and Wales, 96 (11.2%) are known to have died from HTLV-related illness. Deaths most commonly occurred in women (n = 57), persons of black Caribbean ethnicity (n = 47), persons with HTLV-1 (n = 89) and persons with ATLL (n = 53). Median time between HTLV diagnosis and death was 156 days (IQR: 28.5–324.5).

## Discussion

An estimated 22,000 persons are infected with HTLV in England and Wales, yet each year an average of only 86 HTLV diagnoses are made. To explore why there is this discrepancy, we investigated HTLV testing patterns from sentinel laboratories in England. The numbers of persons tested for HTLV increased between 2008 and 2013, and the majority of HTLV tests occurred in secondary care. The biggest proportional increases in testing were seen among women and in primary care services but from a very small baseline, and testing in these settings remains uncommon. Although more testing occurred in men, women are more likely to have a reactive HTLV test and confirmed HTLV diagnosis. 

This paper also updates the epidemiological picture of HTLV provided through enhanced surveillance since it was last described in 2002. The number of persons being diagnosed with HTLV remained constant over the 10-year period. The majority of persons diagnosed were female, of black Caribbean ethnicity and were born outside the UK. HTLV-1 remains the most common form of HTLV diagnosed in England and Wales and most patients were asymptomatic at diagnosis and infected through either heterosexual contact or MTCT. In contrast, the smaller fraction diagnosed with HTLV-2 were more likely to be white, asymptomatic and have a history of injecting drug use.

Our results collected through the enhanced surveillance are consistent with previous descriptions of HTLV-diagnosed persons in England and Wales [12,16], with similar risk factors, particularly by subtype. Compared with Spain, which has conducted similar surveillance, more cases were diagnosed per annum in England and Wales (89 vs 25) [18]. In addition, a higher proportion of cases in England and Wales had HTLV-1 when compared with Spain (90.1% vs 26.1%) and were linked to ATLL (20.5% vs 7.3%), whereas similar proportions were linked to HAM/TSP (England and Wales: 11.9%, Spain: 10.9%).

The vast majority of studies worldwide present data through blood donor testing, antenatal screening and enhanced surveillance, with limited information on general testing patterns. Sentinel surveillance of blood-borne virus testing presents a unique opportunity to gain an insight into where HTLV tests are being requested in England and reviewing changes in testing and prevalence estimates.

Like HIV and hepatitis, HTLV has a long latency period, and a person can be infected for many years before receiving a diagnosis or presenting symptoms. Therefore, it is likely that the vast majority of infections in England and Wales, estimated at 22,000 persons, remain undiagnosed [12]. The introduction of routine HTLV screening of tissue, blood and stem cell donations in 2002, alongside increased disease awareness, may have driven some of the observed increase in HTLV testing. However, the majority of tests still occurred in secondary care specialities where symptomatic patients are more likely to present or where blood, tissue and stem cell donation is common (haematology, renal services and ophthalmology). Therefore, these testing practices are unlikely to impact the undiagnosed proportion. Nevertheless, increased disease awareness, facilitated by the expansion of specialist HTLV clinics from one to four sites in England (London, Birmingham, Manchester and York), will ensure that testing for HTLV will be more widely considered outside of traditional settings.

Our testing data suggests potential missed opportunities for the prevention of maternal transmission of HTLV. While we observed some testing in antenatal, birthing and post-natal units, it is likely that much of this testing is being driven by the screening of tissue for storage and donation, rather than prevention of HTLV transmission, and primary care testing remains infrequent, implying that testing for HTLV infection is uncommon within sexual health services in England.

Prevalence of HTLV in some groups, particularly older individuals and women, was high. Higher positivity rates in older persons are likely to be due to low disease awareness and the long latency period where a person will remain asymptomatic before developing symptoms and being tested. Higher positivity rates in women are probably due to a higher prevalence in women overall, which may result from differences in the efficiency of HTLV transmission between male and female sexual partners, with transmission rates higher from HTLV-positive male partners [8,19]. Work to understand transmission within family settings in Japan estimated transmission rates from men to women to be as high as 60.8%, compared with 0.4% in female-to-male transmission [20], with similar outcomes in studies conducted in Jamaica and the United States [21,22]. In the UK, there are currently no seroprevalence studies with sufficient statistical power to assess differences by sex, however, the prevalence of HTLV in blood donors is higher in women [23]. In addition, these higher positivity rates may reflect differences in the presentation of females to care, with women more likely to present at healthcare settings earlier than men [24-26]. Lima et al. indicated that women in Brazil had faster disease progression than men [27].

The high rate of confirmation of HTLV infection following a reactive result in the initial test supports the high specificity of the currently available assays. This is important as there is concern, particularly in low prevalence regions, that even minimal lack of specificity places a large burden on diagnostic laboratories to investigate many false-negative results. Our finding that more than half of reactive antibody tests were confirmed positive is thus reassuring.

Although 72% of HTLV infections occurred in persons of black Caribbean or black African ethnicity, 94% of diagnoses of ATLL occurred within these ethnic groups. The Caribbean as well as western and southern Africa are endemic regions for HTLV infection and thus there is greater potential for mother-to-child HTLV transmission. HTLV-1 infection acquired perinatally or in infancy through breast-feeding has been associated with risk of ATLL, whereas HTLV-1 infection acquired in England and Wales is more likely to have been due to behaviour during adulthood. These data therefore support the assumption of ATLL risk and also draw attention to the need to consider HTLV-1 infection in any patient with a T-cell malignancy, but especially if they belong to a high-risk ethnic group.

There are two key limitations in our study. The first is the small number of persons with a positive HTLV screen and confirmed HTLV diagnosis. Despite our testing data indicating an increase in testing, with similar proportions testing positive for HTLV, we have not seen the number of persons reported to the enhanced surveillance of HTLV increase over the same time period. In addition, the small number of people diagnosed with HTLV prevented us from looking at demographic and clinical trends. Secondly, we were unable to look at trends in ethnicity in HTLV testing through the SSBBV testing database. It would have been particularly interesting to see whether particular groups are being targeted for testing in line with demographics, with persons of black Caribbean ethnicity representing the majority of those diagnosed with HTLV in England and Wales. Despite these limitations, enhanced surveillance of HTLV has high case ascertainment of persons diagnosed with HTLV in England and Wales, as all confirmatory tests are directed to PHEs VRD, ensuring that our sample is representative.

Since 2013, there have been significant changes to the surveillance of new diagnoses of HTLV, which has resulted in key clinical information being under-reported (< 30% completion rates), making comparison with more recent data problematic. Work is ongoing to improve the completeness of the dataset. However, we do not expect the characteristics of persons newly diagnosed with HTLV between 2014 and 2015 to differ significantly from those diagnosed between 2004 and 2013.

Although characteristics of those with a confirmed diagnosis of HTLV reported to the enhanced surveillance in England and Wales do not appear to have changed over the past 10 years, HTLV testing has increased in England. This increase occurred predominately through increased awareness of HTLV, associated with symptom presentation. Although there have been increases in testing, testing frequency in primary care services, including sexual health services, is still suboptimal and continued work is needed to ensure that HTLV is considered among those at greater risk, tackling the undiagnosed fraction and reducing transmission.
